# Time of day metabolism and endocrine regulation of immune health

**DOI:** 10.3389/fmmed.2026.1982547

**Published:** 2026-10-27

**Authors:** Ashok Hattangady, Virender Singh, Chirag Gala, Narendra Chirmule, Vivek Kadambi

**Affiliations:** 1 Independent Researcher, Bangalore, India; 2 Biopatrika, Haryana, India; 3 National Institute of Pharmaceutical Education and Research Department of Pharmaceutics, Sahibzada AjitSingh Nagar, India; 4 University of Pennsylvania, Philadelphia, PA, United States; 5 Indian Institute of Technology Bombay, Mumbai, India; 6 Belle Sante Institute of Functional Medicine, Bangalore, India

**Keywords:** biomarkers, circadian, endocrine, immune regulation, metabolic

## Abstract

The immune system is a dynamic, energy-dependent network regulated by temporal biology. Advances in circadian biology have enabled the synchronizing of critical immune parameters such as metabolism, endocrine secretions, surveillance, tissue repair, and adaptation to environmental cycles. Here, we propose a metabolomic, and endocrine, chromodynamic model of immune health, based on time-of-day rhythms. Immune resilience is defined by synchronization among three groups of oscillators i) the central circadian clocks, ii) food-entrainable metabolic oscillators, iii) peripheral cellular clocks. The model provides a framework to optimize these interconnected systems to maintain metabolic efficiency, minimize unnecessary inflammation, and enable precise immune responses through temporal gating. External factors of modern lifestyle disruptions, including irregular feeding patterns, sleep deprivation, and chronic stress, induce internal circadian desynchrony, contributing to persistent low-grade inflammation, metabolic dysfunction, immune aging, and chronic non-communicable diseases. We propose that immune health is characterized not merely by the absence of disease, but by stable circadian phase coherence, low inflammatory noise, and adaptive immune resilience. We have listed some chrono-biomarkers of metabolomic and endocrine parameters that may provide a functional assessment of immune resilience, defined by inflammatory tone, immune adaptability, metabolic integrity, and repair capacity. This framework integrates chronobiology, endocrine and immunometabolism to shift medicine from reactive disease management toward proactive assessment and restoration of biological resilience.

## Introduction: *the paradigm from defense to homeostasis*

The immune system has evolved over millions of years to defend the host against pathogens while maintaining tolerance to self-antigens and commensal microbiota. Over the past 50+ years, our understanding of immunity has advanced through a focused mechanistic approach, enabling in-depth characterization of the pathways, cells, proteins, and genes that govern immune responses and contribute to the pathogenesis and treatment of infectious diseases, autoimmune disorders, and cancer (Editors. Immunology is for everyone, 2024). We now know that the healthy immune system is not merely a defensive mechanism but a regulatory system that sustains tissue homeostasis, facilitates repair and orchestrates robust inter-system signaling with minimal auto-inflammatory noise. Significant advances have been made in the understanding the fundamental components of innate and adaptive immunity (Sender et al., 2023). The development of an immune system involves a series of interactions with dendritic cells, lymphocytes, natural killer (NK) cells and other immune cells in lymphoid organs. These interactions include cell-cell combination, induction of cytokines, changes in cell surface receptors, proliferations, and differentiation into effector cells, and immune memory. An effective immune response depends on a balance between activating and regulatory signals, which are influenced by metabolic, endocrine, neurological and several other factors. These factors provide feedback to the nervous and endocrine systems, creating a dynamic bidirectional network that links immune function with the physiological rhythms of the organism. An emerging frontier is chronobiology, which recognizes that immune responses are orchestrated in time as well as space (Skarke et al., 2017; Wyss-Coray and Topol, 2026). Circadian clocks synchronize immune surveillance, metabolism, tissue repair, and host defense with predictable environmental cycles, giving rise to a chrono-dynamic view of immunity. Circadian immunology was first described by adrenergic control of leukocyte trafficking (Scheiermann et al., 2012), which places energy economy at the center of immune timing. In this perspective, we have discussed a framework on recent advances in understanding the homoeostasis in immunology through the lens of a *chrono(logical), dynamic and systemic approach*.

## 50 Years of immunology

*Innate immunity* is the first layer of defense involved in pattern recognition of foreign bodies by delivering biochemical signals with alarm the adaptive system (Carpenter and O'Neill, 2024). *Adaptive immunity* has evolved to provide specificity and memory through specialized cells and receptors generated by the process of clonal selection and gene editing (Carpenter and O'Neill, 2024). *Tissue-specific immunity* has emerged as a new paradigm. In that, the nature of the immune response (to an antigen) is governed by the nature of the local organ-specific immune responses, such that an immune response to an antigen is quite different in the liver, lung, eye or brain (Weisberg et al., 2021). The *microbiome*, influences every aspect of the immune system. And most astoundingly, *neuronal action potentials* - activated in the brain (through thought, experiences, through the 8 senses) - have been shown to influence the (innate) immune system to anticipate (predict) danger (Trabanelli et al., 2025). This aspect of the immune system has been termed as the *anticipatory immune system* (Cohen et al., 2019). Overall, while these systems enable the body to respond and neutralize the plethora of microbes and foreign bodies, the immune system has a vast role in maintaining body homeostasis and “good immune health”.

## Anticipatory chrono-dynamic immune-metabolic framework

The focus in this approach shifts from a *disease-centric* to a *health-centric* view of immunity. Continuous immune activation is metabolically costly (Cohen et al., 2019). To balance effective host defense with energy conservation, the immune system has evolved to operate according to circadian rhythms. Rather than remaining constantly activated, immune cells are deployed, redistributed between the circulation, tissues, and lymphoid organs, and renewed in a predictable daily light and dark cycles that anticipate environmental challenges while promoting tissue repair during rest (Skarke et al., 2017; Scheiermann et al., 2013). This dynamic, energy-efficient strategy, replacing the static view of the immune system is highly sensitive to metabolic cues and shifts in energy utilization can influence their activation, differentiation and longevity a concept now recognized as immunometabolism (Clarke and Mauro, 2026). The systemic approach involved the night/day, or light-and-dark cycles (Figure 1).

**FIGURE 1:**
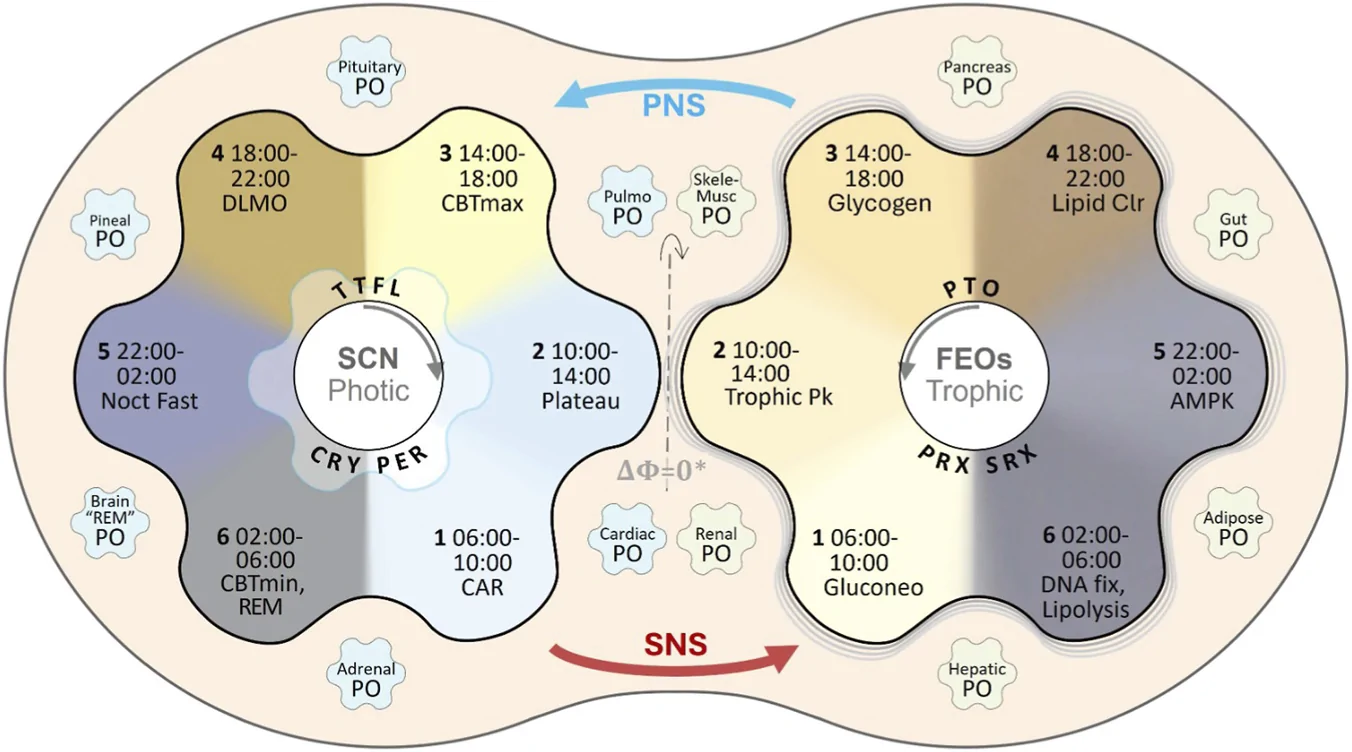
Circadian coordination of metabolomic and endocrine rhythms in immune regulation. Temporal phase‐lock of the circadian i) central suprachiasmatic nucleus (SCN), ii) Food Entrainable Oscillators and peripheral oscillators (FEOs and POs) in tissues, and iii) endocrine and metabolic signals that coordinate immune functions. SCN entrained by photic cues (light and dark) is clocked by genetic transcription‐translational feedback loop (TTFL) driven by the CRY/PER proteins. FEO coordinates processes, including hunger, digestion, satiety, glycogen storage, lipid clearance, glucogenesis, lipolysis and DNA repair. The SCN and FEOs interwork as dual phase‐locked loops with a genetically defined, species-specific offset [Φ (Phi) represents the Phase angle of the FEOs relative to the SCN; Δ (delta) represents the phase deviation from the optimal species-specific baseline]. Optimal baseline alignment is ΔΦ = 0° (diurnal) in humans and ΔΦ = 180° (nocturnal) in mice. Peripheral organs (pituitary, adrenal, pancreas, liver, gut, adipose tissue, kidney, heart, lung and skeletal muscle), contribute synchronism to the network. The sympathetic (SNS) and parasympathetic (PNS) systems provide neural coordination between central and peripheral pathways. The numbered intervals (1‐6) represent successive circadian phases and associated diurnal states. Time‐dependent rhythmic metabolic and endocrine signals regulate immune cell trafficking, endocrine signals, activation thresholds, inflammatory responses and nightly repair. Peripheral systems provide local immune milieu of cells and soluble factors that maintain immune homeostasis. Abbreviations : Light ‐ CAR (Cortisol Awakening Response) and Dark‐ DLMO (Dim Light Melatonin Onset). CRY/PER, (cryptochrome/period, core repressor proteins); signals involved in sleep maintenance and REM-associated neural activity; Core Body Temperature (CBT); Noct Fast (Nocturnal Fasting state).

### Light cycle

It is the surveillance/defense phase. During the light cycle, the immune system prepares the body for anticipated exposure to pathogens and tissue injury. In humans, rising cortisol levels coordinated through the hypothalamic-pituitary-adrenal (HPA) axis under the control of the suprachiasmatic nucleus (SCN), promote the redistribution of leukocytes, including naïve T cells and monocytes. Dimitrov et al. (2009) demonstrated that naïve T cells are negatively correlated with cortisol rhythms. The morning cortisol rise removes naïve CD4^+^ T cells from the blood into bone marrow and lymph nodes through CXCR4 upregulation, while effector CD8^+^ T cells were positively correlated with epinephrine rhythms, increased after low-dose epinephrine infusion, and showed highest expression of beta-adrenergic and fractalkine receptors (CX3CR1). The endocrine hormones, along with circadian cycles, play dual roles in this process by both promoting inflammation and exerting anti-inflammatory effects in immune regulation (Xu et al., 2025). Guided by chemokines, these cells traffic to barrier tissues such as skin, lungs and gastrointestinal tract, and enhance immune surveillance at sites most likely to encounter environmental challenges (Zeng et al., 2024). At the same time, increased endocrine signals, including insulin sensitivity, and thyroid hormones synchronize immune metabolism with the energetic demands of the active phase, favoring rapid innate immune responses and efficient host defense. This *anticipatory* deployment allows rapid detection and containment of pathogens while minimizing unnecessary immune activation.

### Dark cycle

This is the restorative/repair/memory phase. During the dark cycle, the immune system shifts from surveillance to repair and immune restoration. Under the control of the suprachiasmatic nucleus (SCN), increasing melatonin levels from the pineal gland, reduced cortisol levels, enhanced parasympathetic (vagal) activity, and changes in inflammatory mediators promote the migration of circulating leukocytes back to the bone marrow and secondary lymphoid organs (Poole and Kitchen, 2025). Leukocyte trafficking changes during this period, with immune cells redistributing from the circulation toward tissues and lymphoid compartments. Increased interactions among antigen-presenting cells, T cells, and B cells, together with changes in cytokine signaling, support the organization of immune responses and the development and consolidation of immunological memory (Druzd et al., 2017). During sleep, immune cells interact to facilitate antigen presentation, T- and B-cell activation, immune memory formation, and tissue repair (Besedovsky et al., 2019). Cellular maintenance processes, including DNA repair, protein quality control, autophagy, and mitochondrial restoration, are enhanced during this period, while adaptive immune responses are consolidated. In addition to these systemic endocrine signals, immune and tissue-resident cells locally activate hormones such as calcitriol from circulating calcifediol (Bilezikian et al., 2026), providing tissue-specific regulation of immune tolerance, regulatory T-cell differentiation, and barrier homeostasis. Thus, the dark cycle serves as the principal phase for immune restoration, metabolic recovery, and preparation for the next cycle of immune surveillance.

## Integrated timing systems of the immune metabolic-endocrine chronobiome

Fifty years ago, the discovery of the genetic basis of circadian rhythms by Konopka and Benzer (Konopka and Benzer, 1971) followed by the molecular characterization of the transcription-translation-feedback loop (TTFL) involving the BMAL1, CLOCK, PER and CRY by Takahashi (Takahashi et al., 2008) and Reppert (Lee et al., 2001) established the foundation of chronogenetics and ultimately contributed to the recognition of Hall, Rosbash and Young with the 2017 Nobel Prize in Physiology or Medicine.

The human transcriptome consists of Clock-Controlled Genes (CCGs), the expression of which is temporally coordinated through by phase-locking mechanisms that synchronize cellular transcription, metabolism, DNA repair, and immune functions with endogenous circadian and environmental diurnal cycles (Kim and Lazar, 2020; Wang et al., 2022). *The interconnected timing systems* comprise of the i) SCN master clock, ii) the food-entrainable oscillator, and iii) peripheral cellular clocks, together comprise of the oscillatory networks to establish temporal organization of immune function, metabolism, and tissue repair.

1. Suprachiasmatic Nucleus (SCN): The Central Circadian Coordinator

The SCN of the hypothalamus functions as the primary master clock, integrating environmental light signals and synchronizing systemic circadian rhythms through the hypothalamic–pituitary–adrenal (HPA) axis and neuroendocrine pathways (Li et al., 2024). SCN coordinates behavioral, metabolic, and immune rhythms by aligning peripheral clocks with the external day–night cycle (Wyss-Coray and Topol, 2026). It also influences timing of leucocyte trafficking, immune cell activation, cytokine production, and inflammatory responses. This temporal coordination enables immune cells to anticipate predictable changes in environmental exposure and metabolic demand.

2. Food-Entrainable Oscillators (FEOs): *The Nutritional Timekeeper*

Independent of light-driven SCN regulation, food-entrainable oscillators in the liver, hypothalamic nuclei, and gastrointestinal ghrelin-producing cells synchronize circadian programs with feeding–fasting cycles through the gut–brain axis (Petersen et al., 2022). These clocks regulate nutrient sensing, metabolism, and immune energy availability.

3. Peripheral Cellular Oscillators: *The Immuno-metabolomic phase network*

Every nucleated cell contains an autonomous TTFL-based molecular clock, generating peripheral oscillators across the human body (Yi et al., 2022). These cellular clocks function as phase comparators, continuously adjusting local transcriptional programs, including immune activation, metabolic pathways, and tissue repair, in order to maintain systemic circadian alignment.

Thus, immune health may be viewed as a state of *dynamic immune-metabolic endocrine - synchronization* that maintains coherence between central and peripheral circadian clocks. This dynamic process ensures coordination of immune surveillance, metabolism, tissue repair, and adaptation to environmental cues. Circadian disruption converts this highly regulated anticipatory system into a state of chronic metabolic and inflammatory imbalance, characterized by reduced temporal precision, persistent low-grade inflammation, and impaired immune discrimination. Defining health through this chrono-dynamic framework provides a foundation for identifying measurable immune-metabolic endocrine biomarkers that capture the resilience, amplitude, and adaptability of the immune system.

## Endocrine and the clock influences on the immune system

Hormone chronobiology directly dictates immune system behavior through a 24-h master clock in the brain that orchestrates daily peaks and troughs of key hormones such as Cortisol, Melatonin (Figure 2), Human Growth Hormone (HGH), insulin sensitivity (Morris et al., 2015; Van Cauter et al., 1992) and thyroid hormones (Fernandes et al., 1992). This hormonal fluctuation coordinates the migration, activation, and suppression of immune cells to optimize defense against pathogens while preventing autoimmune damage during the day. Endocrine hormonal activity may regulate circadian-dependent movement of lymphoid cells from various lymphoid tissues to peripheral blood. Cortisol, melatonin, HGH, thyroid hormones and insulin sensitivity follow distinct circadian rhythms driven by the brain’s master clock, the SCN. They work in precise opposition to manage energy, sleep quality, and cellular repair (Van Drunen and Eckel-Mahan, 2022; Kim et al., 2015).

**FIGURE 2:**
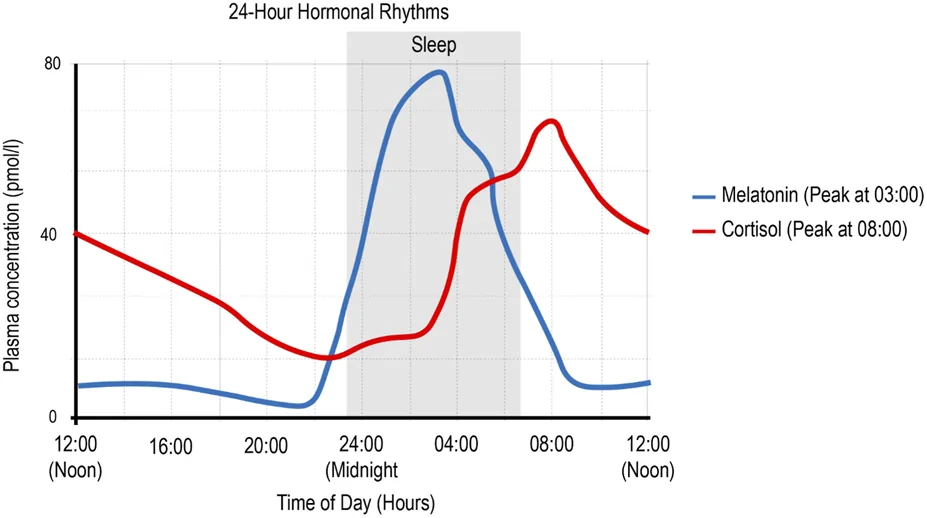
Normal relationship between plasma melatonin and cortisol levels and sleep (shared in grey) in humans across the 24 h of day. Adapted from (Hickie et al., 2013).

The immune system is closely integrated with the endocrine circadian system, which coordinates daily changes in hormone concentrations and the sensitivity of target tissues. During the dark cycle, declining cortisol together with increasing melatonin and growth hormone contributes to a state of immune consolidation and tissue restoration. Cortisol is a potent anti-inflammatory and immunosuppressive hormone (Chen et al., 2026). Its levels naturally decrease to their lowest point in the early hours of the dark cycle, enabling immune activation. Leukocyte trafficking changes during this period, with immune cells redistributing from the circulation toward tissues and lymphoid compartments. Increased interactions among antigen-presenting cells, T cells, and B cells, together with changes in cytokine signaling, support the organization of immune responses and the development and consolidation of immunological memory. The dark cycle therefore provides an important window for immune regulation, repair, and restoration.

The endocrine rhythms are influenced by biological sex, age, and reproductive status. Adrenal hormones, particularly cortisol, exhibit robust circadian patterns, whereas hormones predominantly derived from the gonads show more subtle daily oscillations that are superimposed on longer-term cycles, including the menstrual cycle. Consequently, endocrine regulation of immunity varies across individuals and across the lifespan and should be considered when interpreting chronobiological immune biomarkers and developing chronotherapeutic strategies. The endocrine system functions as a major temporal synchronizer of immunity, coupling the circadian clock to daily changes in immune-cell trafficking, inflammatory activity, metabolism, tissue repair, and immune memory. Disruption of these rhythms—through environmental, behavioral, metabolic, or genetic factors—may impair immune homeostasis and contribute to chronic inflammatory, metabolic, and oncological disease. Understanding and restoring endocrine–circadian alignment therefore represents an important component of a dynamic approach to immune health and disease.

## Chrono-metabolic-endocrine-immune biomarkers

A healthy immune-metabolic system is characterized not by maximal immune activation, but by temporal regulation, including appropriate circadian amplitude, low baseline inflammatory tone, preserved immune reserve, and rapid, proportionate responses to challenge (Szredzka et al., 2025). Therefore, biomarkers of health should capture the resilience, quiescence, and phase coherence of immune–metabolic networks rather than only disease-associated elevations. A panel of circulating markers obtained from routine blood samples can provide an integrated assessment of systemic inflammation, immune adaptability, metabolic homeostasis, tissue repair capacity, and physiological reserve. Importantly, the timing of sampling is critical for many of these biomarkers, since a single measurement may obscure physiologically meaningful diurnal variation. During the examination of impact of diurnal variations in immune responses, Long et al. (Long et al., 2016) reported that morning vaccination favors higher antibody responses. In addition, these studies suggest the influence of time of day on regulation of immune responses. Some examples of biomarkers which span several interconnected dimensions of immune-metabolic and endocrine health include: inflammatory tone (hs-CRP, IL-6, TNF-α, SAA), immune-cell composition and reserve (NLR and naïve T cells), adrenal and circadian regulation (cortisol and DHEA-S), nocturnal restoration (melatonin and GH), and endocrine-metabolic regulation (thyroid hormones and vitamin D metabolites). Dimitrov and colleagues have shown that the morning cortisol rise removes naïve T cells from the blood into bone marrow and lymph nodes through CXCR4 upregulation, while adrenaline mobilizes effector CD8 cells and NK cells (Dimitrov et al., 2009). Besedovsky, Lange and Haack (Besedovsky et al., 2019) demonstrated that effector cells and neutrophils rise in the blood during the active phase; naïve and central memory T cells peak at night. The greatest value of such biomarkers may not lie in any individual measurement, but in the relationships between them and their temporal patterns.

We have previously reviewed literature on the concept of an ImmunoSCORE as a signature to monitor the evolution of an immune responses to infectious agents, autoimmunity, cancer and therapeutic biologics (Gopallawa et al., 2026). Monitoring a dynamic immune response will require multiple immune signatures over the course of time and organ(s). The advent of systems immunology will enable the understanding of the complexity of multi-dimensional aspects of the immune system. The emerging opportunity is therefore to move from a static biomarker model to a dynamic immune-metabolic phenotype. Serial measurements obtained at defined biological times, combined with clinical, metabolic, behavioral, and physiological data, could quantify the amplitude, phase, coherence, and recovery of immune–endocrine networks. Such an approach could ultimately define immune-metabolic resilience, the capacity of the organism to anticipate physiological challenges, mount an appropriate immune response, and efficiently return to homeostasis.

### Integration: a chrono-dynamic definition of immune health

The immune system is not a static defense mechanism but a dynamic, temporally organized network that has evolved to anticipate environmental challenges while preserving energy and tissue integrity. The integration of circadian clocks, metabolic sensors, and peripheral immune oscillators creates a highly coordinated immunometabolic ecosystem with remarkable precision. Disease emerges when this temporal architecture loses synchronization, resulting in chronic inflammatory noise, impaired metabolic flexibility, and diminished repair capacity. A new definition of health therefore requires moving beyond measurements of disease burden toward assessment of biological coordination and resilience. A healthy organism maintains robust circadian phase coherence, appropriate immune activation thresholds, metabolic harmony, and efficient restoration during periods of rest. Chrono-biomarkers provide an opportunity to quantify this state by measuring the amplitude, adaptability, and stability of immunometabolic regulation. This chronodynamic perspective reframes health as an active biological state of synchronized resilience, where the goal of medicine shifts from suppressing pathology alone to restoring the evolutionary programs that maintain immune balance, metabolic integrity, and lifelong adaptability (Man et al., 2016).

With the advent of multi-omics, organ-specific data, and systems immunology, it is possible to evaluate this chronodynamic systems framework across diverse disease states and determine how disruptions in temporal immune organization contribute to pathology. Future studies should investigate whether non-infectious chronic diseases, including obesity, diabetes, cardiovascular disease, neurodegeneration, and cancer, are associated with distinct patterns of chronobiological immune dysfunction. Similarly, chronic infections, autoimmune diseases, allergic disorders, and immune aging may reflect persistent alterations in immune-metabolic synchronization rather than isolated molecular defects. Integrating longitudinal multi-omics, wearable physiological monitoring, circadian biomarkers, and computational modeling will enable quantitative characterization of these dynamic immune states. In this respect, Skarke et al. have recently reported studies of analysis of large datasets from the United Kingdom biobank, on the chrono biome in young and old apparently healthy individuals (Skarke et al., 2026). Such studies may identify disease-specific immune based chronodynamic signatures, uncover novel therapeutic windows for chronotherapy, and provide a framework for precision medicine based on restoration of temporal immune homeostasis rather than treatment of static pathological endpoints.
